# The Use of Pericapsular Nerve Group (PENG) Block in Hip Surgeries Is Associated With a Reduction in Opioid Consumption, Less Motor Block, and Better Patient Satisfaction: A Meta-Analysis

**DOI:** 10.7759/cureus.28872

**Published:** 2022-09-06

**Authors:** Anwar U Huda, Hashsaam Ghafoor

**Affiliations:** 1 Anesthesia, Security Forces Hospital Program, Riyadh, SAU; 2 Anesthesia, Hamad Medical Corporation, Al Khor, QAT

**Keywords:** vomiting, pain, peng block, nausea, hip surgery

## Abstract

Pericapsular nerve group (PENG) block has been successfully utilized as an optional regional anesthesia approach to manage the pain for hip surgeries without affecting motor function. During recent years, the applications of PENG block are expanding. There is one previous review on PENG block for hip surgeries in the scientific literature and it is limited to case series and case reports only. We found few randomized controlled trials related to the role of PENG block in recent literature. So, a meta-analysis was done to evaluate the role of PENG block in managing postoperative pain after hip surgeries.

We followed PRISMA guidelines to perform this meta-analysis. Online databases including Medline and ScienceDirect were used. This review was registered with the PROSPERO database (CRD42022297694) in January 2022. The included studies in this review reported opioid use, pain control after surgery, and side effects associated with PENG block among patients undergoing hip surgeries. The Review Manager software, i.e. RevMan for Mac 5.4 (Cochrane Collaboration, Oxford, UK) was utilized to conduct a meta-analysis.

During this meta-analysis, six randomized trials were included. Our results demonstrated that PENG block usage for patients undergoing hip surgery is correlated with a significant reduction in opioids in the first 24 h after surgery (p=0.05). It also resulted in significant prolongation of time to first request analgesia with mean difference as 3.82 h (0.05-7.60), (p=0.05). Our results showed that PENG block is associated with better patient satisfaction as well. The PENG block resulted in less motor block in the postoperative period (p=0.0002). In conclusion, PENG block can significantly reduce 24-h opioids consumption after hip surgery. This block also resulted in prolonged time to first request of analgesia postoperatively. There is less risk of motor block and hence the potential for better physiotherapy.

## Introduction and background

The ultrasound guided peripheral nerve blocks including fascia iliaca block (FIB), femoral nerve block (FNB), and lumbar plexus block are commonly utilized opioid-sparing techniques for hip surgeries [1-4]. The anterior hip capsule is innervated through articular branches of the femoral nerve, obturator nerve, and accessory obturator nerve as confirmed through prior anatomic studies, signifying that all these nerves must be the key targets regarding hip pain control that may be blocked through PENG block [5].

The PENG block is an innovative technique performed under ultrasound guidance. It was described in 2018 by Girón-Arango and co-workers to block the articular branches of femoral nerve, obturator nerve, and accessory obturator nerve [2, 6-8]. It has been successfully utilized as regional anesthesia approach to manage the pain for hip surgeries without affecting motor function [9-12]. Several studies have been conducted about effectiveness of PENG block in pain control of hip fracture; however, further investigations are also required to ascertain its role [13]. We could only identify one previous review on PENG block for hip surgeries although it included case reports and case series only [8]. There have been few randomized controlled trials recently. This meta-analysis was carried out to assess the impact of PENG block on analgesia and opioid use after hip surgeries as compared with other blocks or no block.

## Review

Methods

This systematic review of scientific literature was carried out to elucidate the PENG block usage for hip surgery. The PRISMA guidelines were followed while utilizing the online databases such as ScienceDirect and Medline. This review was registered with the PROSPERO database (CRD42022297694) in January 2022. Literature searches were performed by two authors in November 2021 and repeated in December 2021 in order to assure the accuracy. A search plan utilized in the ScienceDirect and Medline is demonstrated in Table 1.

**Table 1:** Search strategy for Medline. PENG, pericapsular nerve group

Search term	Number of studies
PENG block	3186
PENG	90
#1 OR #2	3210
Hip surgery	92135
#3 AND #4	66

All related randomized trials were enrolled in this study that were printed in English. All studies reported opioid use, pain control after surgery, and side effects among patients who received PENG block for hip surgery. All approaches for performing PENG block were accepted if clearly mentioned in the methodology. The studies which described one of the two primary outcome measures including early postoperative pain scores or opioid use after surgery were included. The early postoperative period was defined as the first 24-h after the surgical treatment. Secondary outcome measures were the occurrence of nausea and vomiting, dizziness, drowsiness, and pruritus during 24-h after surgical treatment. For our review, we only included primary research and hence, review articles, abstracts, and comments were not included. To collect the reference data, a pre-specified table was used regarding populations and outcomes from the individual studies. The data were gathered such as general details of studies (name of the Journal, publication year, study design, outcome measures, and groups included), sample size, study participants, intervention (dosages and timing of administration), and outcomes (opioids use, pain score, and adverse events). Revised Cochrane risk-of-bias tool for randomized trials (RoB 2) was utilized for the assessment of risk of bias independently by two authors. Consolidated Standards of Reporting Trials checklist was used to appraise the individual studies. During the review, all discrepancies were solved by discussion between both authors. 

We used the Review Manager software (RevMan for Mac, version 5.4; Cochrane Collaboration, Oxford, UK) to conduct a meta-analysis of all studies which was included in this review. The data heterogeneity was evaluated by measuring I2. The data on the pain scores at various time points and 24 h opioid consumption were pooled. While adverse effects including postoperative nausea and vomiting, dizziness, drowsiness, and pruritus were pooled devoid of any time point and recorded as being either present or absent. Mean difference/standardized mean difference was measured for continuous data to report the treatment effect while odds ratios were measured regarding dichotomous data in our review. A random-effect model was utilized during this meta-analysis and level of significance was set by using p-value ≤ 0.05.

Results

**Figure 1:**
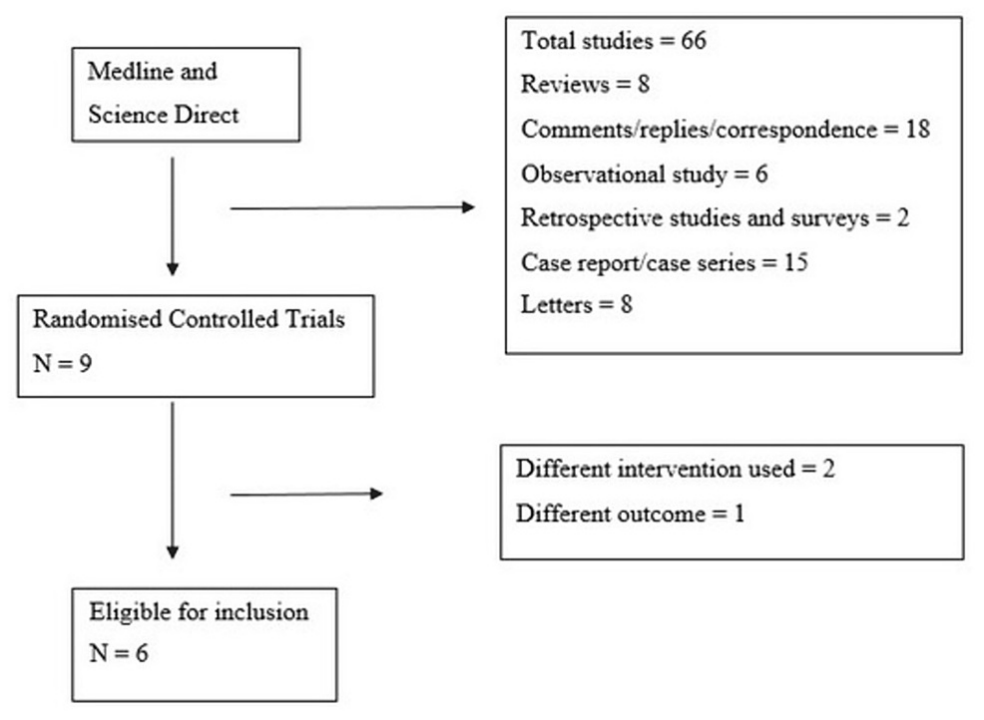
Flow diagram for search process.

Sixty-six studies were identified from this search plan in ScienceDirect and Medline. We included six randomized trials (n=346) in our review after thoroughly evaluating all studies as demonstrated in Figure 1.

A brief detail of all included studies is shown in Table 2. We did evaluation of bias in all studies utilizing RoB 2. All the studies were found to have a low risk of bias.

**Table 2:** Concise details of included studies. FIB, fascia iliaca block; PENG, pericapsular nerve group; FNB, femoral nerve block; NRS, numeric rating scale; PROMs, patient-reported measures outcomes; VAS, visual analog scale

Study	Population	Intervention(s)	Comparator	Outcome	Results
Aliste et al, (2021) [6] N=40	ASA I-III elective unilateral total hip arthroplasty	PENG block, with 20 mL of levobupivacaine 0.5% with epinephrine 5 μg/mL	Ultrasound-guided FIB with 40 mL of levobupivacaine 0.25% with epinephrine 5 μg/mL	Primary: quadriceps motor block 6 h postoperatively; secondary: block performance time block-related adverse events static and dynamic pain score	Intervention group (PENG block). Lower incidence of quadriceps motor block. Better preservation of hip adduction. Decreased sensory block of the anterior, lateral, and medial thighs. No clinically significant intergroup differences were found in terms of postoperative pain scores, cumulative opioid consumption at 24 and 48 h, ability to perform physiotherapy, opioid-related side effects, and length of hospital stay
Lin et al. (2021) [14] N=60	ASA I-IV unilateral total hip arthroplasty	PENG block with 20 mL of 0.75% ropivacaine	FNB with 20 mL of 0.75% ropivacaine	Primary: pain scores (NRS) 0-10 at recovery unit (day 0) at 4 h postoperatively secondary: NRS pain scores on day 1 postoperative quadriceps strength. Perioperative opiate use. Postoperative complications. Patient satisfaction length of hospital stay. PROMs.	Intervention group (PENG block) PENG group experienced less pain 63% experienced no pain, 27% mild pain, 10% moderate to severe pain. FNB group 30% reported no pain, 27% mild pain, 36% moderate to severe pain. NRS pain scores on day 1 similar between both groups quadriceps strength 60% intact in the PENG group vs. none intact in the FNB group. Postoperative opiate use was similar between both groups. Complication rates were similar between both groups. Patients were more satisfied with the analgesia received in the PENG group (97%) vs. (70%) in FNB group. PROMs were similar between groups
Mosaffa et al. (2021) [13] N=52	ASA I-II primary hip fracture	PENG block with ropivacaine 0.5% 3 mL/kg.	FIB ropivacaine 0.5% 3 mL/kg	Primary: VAS score was assessed before blocks procedure (baseline). VAS 15 min after blocks (before spinal anesthesia) VAS in the sitting position for spinal anesthesia VAS in recovery room. VAS at 6 and 12 h after surgery. Secondary: The first time of the analgesic consumption after surgery. Total dose of morphine consumption during 24 h. Side effects between two groups.	No significant difference between VAS before blocks procedure between two groups (baseline). After 15 min of blocks and after 12 h of post-surgery, VAS score significantly reduced in the PENG block group compared with the FIB group. The first time of the analgesic consumption after surgery was significantly longer in the PENG block compared with the FIB. The total dose of morphine consumption during 24 h significantly reduced in the PENG block as compared to FIB. No significant differences between side effects between two groups.
Scanaliato et al. (2020) [15] N=64	Age more than 16 and less than 50 years for hip arthroscopy	Pericapsular anesthetic injection 30 mL of ropivacaine and 12 mg of morphine	Lumbar plexus blockade 40 mL of 0.375% ropivacaine with 4 mg of preservative-free dexamethasone	Primary: pain numeric rating scale Secondary: Time to discharge from the recovery room Morphine equivalents received in the recovery room 10 mg immediate-release oxycodone taken by the patient in the first 48 hours Patient satisfaction with postoperative analgesia, Adverse effects	No significant differences in pain at all time in the recovery room. Time to discharge from the recovery room did not vary between treatment groups. Home analgesia during the first 48 h, as measured by morphine equivalent dose, did not vary between treatment groups. Percentage of patients satisfied with their pain control did not vary between treatment groups.
Pascarella et al. (2021) [10] N=80	ASA I-III primary unilateral total hip arthroplasty age 16 and above	PENG block with 20 mL of ropivacaine 0.375%	No block (control group)	Primary: The main outcome was postoperative pain, assessed using a NRS*** at 12, 24 and 48 h after total hip arthroplasty. Secondary: Postoperative opioid consumption; Patient mobilization assessment length of stay Presence of any adverse effects (nausea and vomiting)	The maximum pain score of patients receiving the pericapsular nerve group block was significantly lower than in the control group at all time-points. Pericapsular nerve group showed a significant reduction in opioid consumption, better range of hip motion and shorter time to ambulation. No significant difference in hospital length of stay. No difference of adverse effect (nausea and vomiting).
Zheng et al. (2021) [12] N=70	Primary total hip arthroplasty age 18-70 years	PENG block with 20 mL 0.5% ropivacaine	20 mL 0.9% saline (placebo group)	Primary: highest pain score reported in the recovery room. Secondary: quadriceps strength. Pain scores. Opioid use. Opioid-related side effects up to 48 h after surgery.	Highest pain scores in the recovery room were significantly different from each other more in placebo group than PENG. No differences between the two groups’ highest pain scores after recovery room discharge. No significant between-group differences in quadriceps strength levels. No differences in the two groups’ pain scores at rest. Intraoperative opioid consumption lower in the PENG group than in the placebo group. Similar levels of rescue opioid use during recovery room stay and 48 h after their recovery room discharge. The incidence of nausea was comparable between the groups, but the incidence of vomiting was reduced in the PENG group.

Pain scores

From the included studies, Aliste et al. [6] did not find any significant difference in their study regarding pain scores during first 24 h between PENG block and fascia iliaca block (FIB) groups. Lin et al. [14] showed that pain scores in the post anaesthesia care unit (PACU) were significantly different between PENG and FNB (p=0.04). Out of 30, 19% (63) patients in PENG group vs. 9 (30%) patients in FNB group experienced no pain in PACU. Although, they did not find any significant difference in pain scores at other time points. A study carried out by Mosaffa et al. [13] found insignificant difference in pain scores between PENG block and FIB in early postoperative period although pain score was found significantly lower among patients in PENG group at 12 h when compared with FIB, 3.01 (1.08) vs. 3.91 (1.48), p=0.021. Pascarella et al. [10] reported that maximum numeric rating score of pain in PENG block was significantly lower when compared with patients in no block cohort at all-time points after surgery (p<0.001). Scanaliato et al. [15] in their study demonstrated insignificant difference in the pain scores during first 90 min postoperatively between PENG block and lumbar plexus block groups. Zheng et al. [12] reported that highest pain scores in the PACU were significantly different between PENG and placebo group with -1.9 difference (p<0.01). There was no difference in mean and highest pain scores of patients in both cohorts at other times.

From the included studies, a pooled meta-analysis demonstrated that the pain scores were not significantly different between the PENG block and other cohorts in PACU and at 6, 12, and 24 h after surgery [(p=0.59, confidence interval, CI: -0.38, 0.22), (p=0.10, CI: -2.13, 0.17), (p=0.18, CI: -2.98, 0.55)] respectively.

Time to first request analgesia

Mosaffa et al. [13] in their study reported that the time to first request analgesia in PENG group was significantly lengthier when compared with patients in FIB group, 4.7 +3.1 vs. 2.58 + 2, (p=0.007). Pascarella et al. [10] indicated that time to first request analgesia in PENG block group was 6 h longer than the control group, 12+6.7 vs. 6+4.9 (p=0.001).

**Figure 2:**
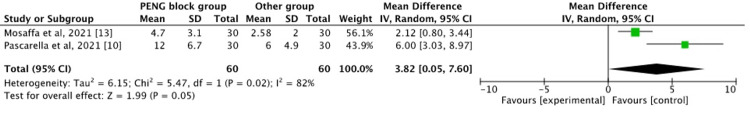
Forest plot for time to first request analgesia.

A pooled meta-analysis of the two included studies demonstrated a significant difference in the time to first request analgesia during the postoperative period with the use of PENG block, mean difference of 3.82 h (p=0.05) as shown in Figure 2. 

Opioid consumption

A study carried out by Aliste et al. [6] reported that 24 h opioid consumption was not significantly different between PENG block and FIB, 4.8 + 5.3 vs. 4.5 + 4.7, (p=0.85). Lin et al. [14] were also unable to find any statistically significant difference in 24 h opioid consumption between PENG block and FN block (p=0.59). Mosaffa et al. [13] indicated a significant difference in 24 h opioid consumption between PENG block as compared to FIB, 54 + 25.67 vs. 74.37 +18.87 mg, p=0.008. Pascarella et al. [10] also reported a significant difference in 24 h morphine-equivalent opioid consumption between PENG block and no block group, 4 + 4.5 vs. 8.9 + 4 mg (p<0.001). Zheng et al. [12] found insignificant difference in opioid consumption during 48 h postoperative period with mean difference as 3.5 (-16.4 to 9.4) (p=0.59).

**Figure 3:**
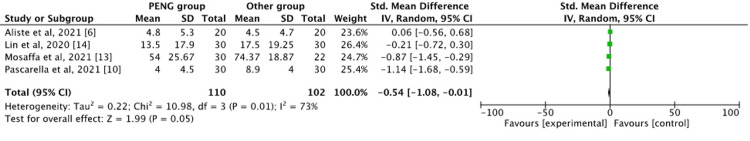
Forest plot for opioid consumption.

From the included studies, a pooled meta-analysis showed a significant decrease in opioids dose consumption during the first 24 h after surgery with the use of PENG block, standardized mean decrease of 0.54 mg (p=0.05) as shown in Figure 3.

Satisfaction level

Lin et al. [14] demonstrated that patients in PENG group were more satisfied compared to patients in FNB group. Out of 30 patients, 29 (97%) in PENG group were satisfied compared to 21 (70%) in FNB while 1 (3%) was ambivalent in PENG vs. 9 (27%) in FNB group, p=0.02. Scanaliato et al. [15] showed that out of 32, 30 (94%) patients in PENG block group were satisfied compared to 29 (91.44 %) in lumbar plexus block group while the difference was not found as statistically significant (p=0.65). Zheng et al. [12] found insignificant difference in mean satisfaction score between PENG block and placebo cohort, 10 + 1 vs. 9 + 1, p=0.08.

**Figure 4:**
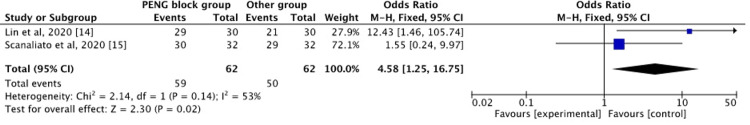
Forest plot for postoperative satisfaction.

A pooled meta-analysis of the included studies demonstrated that the use of PENG block resulted in a significantly better satisfaction level during the postoperative period (p=0.02), as shown in Figure 4. 

Motor block

Aliste et al. [6] found that 19 (95%) patients in PENG block reported no motor block at knee extension at 24 h postoperatively vs. 13 (65%) patients in FIB though it was found statistically insignificant (p=0.102). Lin et al. [14] in their study showed that 27 (90%) patients in PENG block vs. 15 (50%) patients in FNG reported intact quadriceps strength on day 1, p=0.004. Pascarella et al. [10] found better range of motion in PENG block group compared to control group, 62.3 + 20.2 degrees vs. 38.7 + 22.4 degrees respectively (p<0.001). Also, time to first walk was found significantly shorter in PENG block group than in control group, 22.1 + 9.6 vs. 32.4 + 10.6 h respectively (p<0.001). Regarding quadriceps strength level, Zheng et al. [12] found insignificant difference between PENG block and placebo group. 

**Figure 5:**
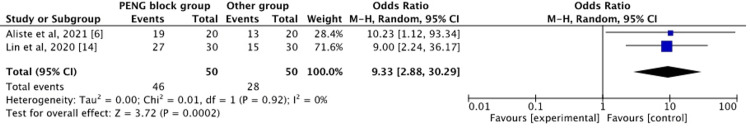
Forest plot for postoperative motor block.

A pooled meta-analysis of included studies demonstrated a significant difference in incidence of motor block postoperatively in PENG block group compared to other cohorts (p=0.0002), as shown in Figure 5.

Postoperative nausea and vomiting

In the study by Aliste et al. [6], two (10%) patients experienced PONV in PENG block group compared to none in FIB group. Pascarella et al. [10] found that one (3%) patient in PENG block group vs. 3 (10%) in control group experienced postoperative nausea and vomiting. Scanaliato et al. [15] showed that 10 (31%) patients felt nausea in PENG block group compared to seven (22%) patients in lumbar plexus block group.

From the included studies, a pooled meta-analysis showed insignificant difference in postoperative nausea and vomiting between PENG block and other groups, 12.9% incidence in PENG group vs. 20.3% in control group, p=0.26.

Discussion

The current meta-analysis and systematic review demonstrated that the use of PENG block could be a preferred option as a regional block in hip surgical treatments. The PENG block reduced postoperative opioid consumption in the first 24 h after hip surgeries. It also prolonged the time to first request of analgesia. It is found to have lesser risk of motor block in postoperative period which is usually associated with early walking and ease of physiotherapy. Also, PENG block resulted in better patient satisfaction postoperatively.

There is growing evidence that enhanced recovery after surgery (ERAS) pathways implementation can decrease the hospitalization time, reduce complications, and is inexpensive for patients experiencing hip surgical treatment [16]. Also, this multimodal pathway containing peripheral nerve block could have considerable impact on rare complications as well as perioperative results after orthopedic main surgery. Compared to conventional IV opioids in postoperative early period, the peripheral nerve block can enhance dynamic pain control, hasten the recovery, and decrease the usage of opioids and associated unfavorable outcomes [17-19]. A Cochrane review demonstrated that regional anesthesia use can reduce the postoperative complications risk. It also resulted in earlier mobilization and decreased opioids consumption in hip fractures [20]. The PENG block was initially utilized like an alternative regional anesthesia technique for pain control after hip fractures although its applications and indications are expanding [21-24]. According to the review by Del Buono et al, the most of PENG block were performed for analgesia related to hip e.g., hip fractures, pelvic fractures, hip surgery, etc. [8]. Girón-Arango et al. in his case series found a seven-point reduction in pain score after PENG block in hip fractures [25]. Almost similar results were demonstrated in other case series as well [26-29]. Guay et al. in a Cochrane systematic review showed a 3.4-point reduction in pain score by the use of nerve block in hip fractures [20]. Lim et al. in one retrospective study on elderly hip fracture patients showed that pain score at 6 h postoperatively in regional nerve block group was significantly lower compared to control cohort, 2.8 ± 1.5 vs. 3.3 ± 1.6, respectively (p=0.03) [30]. However, insignificant difference was found in pain scores at 12 and 24 h after surgery. In contrast, our meta-analysis found insignificant difference in pain scores in PACU and at 6, 12, and 24 h postoperatively with the use of PENG block compared to other group in patients undergoing hip surgery.

Del Buono et al. in their review described the PENG block as an opioid sparing analgesic strategy for hip analgesia [8]. Our meta-analysis results also demonstrated a significant decrease in opioid use during 24 h postoperatively with the PENG block use when compared with other groups with mean difference in morphine equivalent doses of 4.92 mg (p=0.05). Although, Lim et al. [30] did not show any significant difference in opioids consumptions for two postoperative days with the use of peripheral nerve blocks. Giron et al. [25] in their study demonstrated the better preservation of quadriceps muscle power in postoperative period with the use of PENG block when compared with FNB. This can be explained by the observation that this block involves only the articular branches of the femoral nerve, obturator nerve, and accessory obturator nerve. Hence, it does not block the femoral nerve motor branches that innervate the quadriceps muscles. This finding is similar to what was found in the study by Short et al. [31] as well. Ghodki et al. [32] showed that a normal quadriceps motor activity was found in only 13% of patients at 12 h postoperatively in patients receiving FNB. Our meta-analysis also highlighted a significantly decreased motor block of quadriceps muscle with the use of PENG block compared to other blocks. Although, some studies mentioned a complete FNB and obturator nerve block after PENG block [33-36]. Yu et al. described two cases of quadriceps muscle weakness resulting in inability to perform a straight leg raise following PENG block [21].

Several limitations were observed in this meta-analysis. Firstly, the doses and timings of PENG block were different. Aliste et al. [6] performed PENG block postoperatively in PACU using 20 mL of 0.5% levobupivacaine with 5 mcg/mL of epinephrine. Lin et al. [14] utilized 20 mL of 0.75% ropivacaine for PENG block prior to induction of anesthesia. In the study by Mosaffa et al. [13], 3 mL/kg of 0.5% ropivacaine was used for the block pre-induction. PENG block was carried out after spinal anesthesia and prior to surgical incision using 20 mL of 0.375% ropivacaine in the study by Pascarella et al. [10]. Scanaliato et al. [15] did PENG block after anesthesia induction using 30 mL of ropivacaine and 12 mg morphine. Zheng et al. [12] performed block preoperatively using 20 mL of 0.5% ropivacaine. Secondly, type of anesthesia used in different studies was also different. General anesthesia was used in studies carried out by Scanaliato et al. [15] and Zheng et al. [12] while spinal anesthesia was performed in studies conducted by Aliste et al. [6], Mosaffa et al. [13], and Pascarella et al. [10]. In the study by Lin et al. [14], both anesthesia types were used depending on discretion of primary anesthesia team. Another limitation of our meta-analysis was different comparison groups. In studies by Pascarella et al. [10] and Zheng et al. [12], comparison was made between PENG block group and control group (no block). While in other studies, PENG block cohort was compared with another block group. Aliste et al. [6] and Mosaffa et al. [13] compared PENG block with FIB, while Lin et al. [14] used FNB and Scanaliato et al. [15] used lumbar plexus block for comparison. Another limitation is that none of the study reported any re-admission or prolonged hospital stays due to inadequate analgesia, nausea, and vomiting. 

## Conclusions

The PENG block can significantly reduce 24 h opioids consumption after hip surgery. Time to first request of analgesia postoperatively is also prolonged with the use of PENG block. There is less risk of motor block and hence better physiotherapy. The PENG block is also associated with better patient satisfaction postoperatively.
